# Primary carcinoma of the *rete testis* in a Nellore bull

**DOI:** 10.1007/s11259-026-11299-w

**Published:** 2026-05-27

**Authors:** Nayne Vieira da Silva, Renato Lima Santos, Tatiane Furtado de Carvalho, Nayara Ferreira de Paula, Matheus Franca Pereira, Ricardo Araújo Micai, Gabriella Faria Pereira, Diego José Zanzarini Delfiol, Mônica Horr, Hugo Shisei Toma, Geison Morel Nogueira

**Affiliations:** 1 Postgraduate Program in Veterinary Sciences, Federal University of Uberlândia, Uberlândia, MG Brazil; 2 Department of Veterinary Clinics and Surgery, School of Veterinary Medicine, Federal University of Minas Gerais, Belo Horizonte, MG Brazil; 3 Animal Pathology Laboratory, School of Veterinary Medicine and Animal Science, Federal University of Uberlândia, Uberlândia, MG Brazil; 4 School of Veterinary Medicine and Animal Science, Federal University of Uberlândia, Uberlândia, MG Brazil; 5 School of Veterinary Medicine and Animal Science, Federal University of Uberlândia, Av. Mato Grosso No. 3289 – Block 2S. Umuarama District, Uberlândia, MG 38405-314 Brazil

**Keywords:** Cattle, Mediastinum testis, Carcinoma, Cystic dilation, Immunohistochemistry, *Rete testis*

## Abstract

Carcinoma of the *rete testis* is an extremely rare neoplasm, which has not been previously reported in cattle. Therefore, the aim of this report is to describe a case of carcinoma of the *rete testis* in a bull, including clinical, ultrasonographic, histopathological, and immunohistochemical findings. An eight-year-old Nellore bull was referred for evaluation following a history of hemospermia. In the ultrasonographic evaluation, dilation of the left mediastinum testis was observed, as well as the presence of anechoic cystic structures. Unilateral orchiectomy was performed on the affected testis, which was subsequently submitted for histopathological and immunohistochemical examination. Microscopically, there was a neoplasm in the *rete testis*, unencapsulated, poorly demarcated, and infiltrative, but restricted to the mediastinum testis. Neoplastic cells had marked and diffuse immunostaining for vimentin and cytokeratin but did not stain for CD31. Based on the anatomic location of the lesion restricted to the mediastinum testis and adjacent to the *rete testis* epithelium, the histopathologic features, and the immunohistochemical expression of vimentin and cytokeratin by the neoplastic cells, a diagnosis of carcinoma of the *rete testis* was established. Therefore, it is important to include this neoplasm as a rare differential diagnosis in bulls presenting with hemospermia or intratesticular cystic alterations.

## Background

Testicular tumors in bulls are uncommon. The most frequently reported types include sustentacular (Sertoli) cell tumors (Vissiennon et al. 2016), interstitial (Leydig) cell tumors (López et al. 1994), seminomas (Foster 2007), and less frequently, teratomas (Jacinto et al. 2021), yolk sac carcinomas (Sakaguchi et al. 2013), embryonal carcinomas (Agnew and MacLachlan 2017) and mesotheliomas (Sutton 1988). Clinical signs are generally nonspecific and include non-painful testicular enlargement, occasionally accompanied by scrotal asymmetry or reduced fertility. Ultrasonographically, most intratesticular tumors appear as solid, hypo- or heterogeneous masses, while gross examination may reveal firm, pale or multinodular lesions. Histologically, each tumor type exhibits characteristic morphological features (Foster 2007; Agnew and MacLachlan 2017).

Carcinoma of the *rete testis* is an extremely rare neoplasm, with cases documented primarily in humans, horses, dogs, sheep, and rodents (Radi et al. 2004; Agnew and MacLachlan 2017; Imaoka et al. 2022; Petrović et al. 2023). Clinical findings are generally nonspecific, with limited evidence regarding predisposing factors. In animals, available data is restricted to isolated reports, without reproducible patterns for age, laterality, hydrocele, or hemospermia. On ultrasonography, lesions tend to be centered in the mediastinum, sometimes exhibiting cystic or solid components. Grossly, these tumors typically expand the mediastinum and may display papillary, tubular, or solid patterns. Histologically, they originate from the *rete testis* epithelium and may show variable architectural patterns with varying degrees of cytological atypia (Radi et al. 2004; Agnew and MacLachlan 2017; Imaoka et al. 2022).

Differential diagnoses for *rete testis* tumors include neoplasms with overlapping morphological features, such as malignant mesothelioma of the vaginal tunics, sustentacular cell tumors, seminomas and teratomas located near the mediastinum, as well as inflammatory and infectious conditions. Definitive diagnosis includes confinement of the lesion to the mediastinum testis without extension to the parietal tunic, identification of a transition from normal *rete testis* epithelium to neoplastic epithelium, and the exclusion of morphologically similar neoplasms. Histological features must be incompatible with other tumor types, while immunohistochemistry further supports the diagnosis by excluding alternative differential diagnoses (Radi et al. 2004; Chekol and Sun 2012; Agnew and MacLachlan 2017; Petrović et al. 2023).

The aim of this report is to describe a case of carcinoma of the *rete testis* in a Nellore bull *(Bos indicus)*, detailing the clinical presentation, ultrasonographic findings, gross and histopathological features, and immunohistochemical profile.

## Case presentation

### History and clinical findings

An eight-year-old Nellore bull was referred to the veterinary hospital due to persistent hemospermia, observed in eight semen collections at a breeding center. The intervals between sample analyses ranged from two to 46 days.

**Fig. 1:**
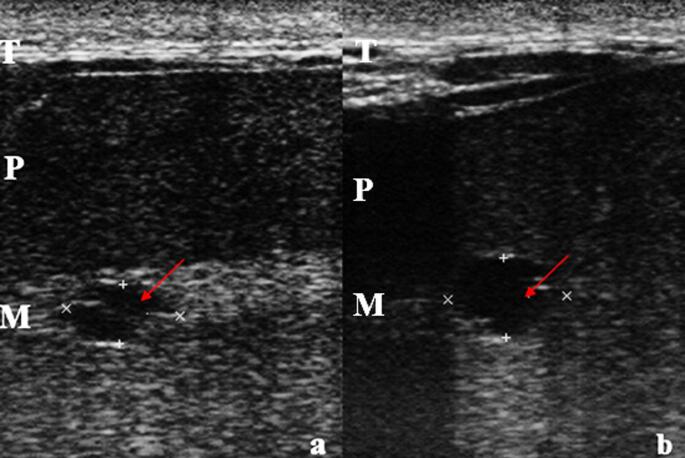
Ultrasonographic images of the mid-third of the left testis in the longitudinal plane obtained five days prior to unilateral orchiectomy, showing two anechoic cystic dilations of the mediastinum. (T: Dartos muscle / P: Parenchyma / M: Mediastinum / Arrows: cystic dilation). (**a**) distal structure − 0.73 cm x 1.39 cm. (**b**) proximal structure − 0.97 cm x 1.45 cm

During clinical examination, increased firmness was noted upon palpation of the entire surface of the left testis, compared with the right testis, which had a firm-elastic consistency. No pain, abnormal motility, or asymmetry between the testis was observed. Complementary diagnostic tests included complete blood count, renal and hepatic clinical biochemistry, urinalysis, and bilateral testicular ultrasonography. Ultrasonography revealed dilation of the left mediastinum testis and two anechoic cystic structures (Fig. 1).

The laboratory results were otherwise unremarkable. However, due to the history of persistent hemospermia and the ultrasonographic findings, unilateral left orchiectomy was performed. Following the procedure, the testis was sent for histopathological examination.

Twenty days after surgery, the animal was discharged and returned to the breeding center. Serial semen collections over four months showed progressive improvement in semen quality, reaching 54% sperm motility.

### Gross pathology

**Fig. 2:**
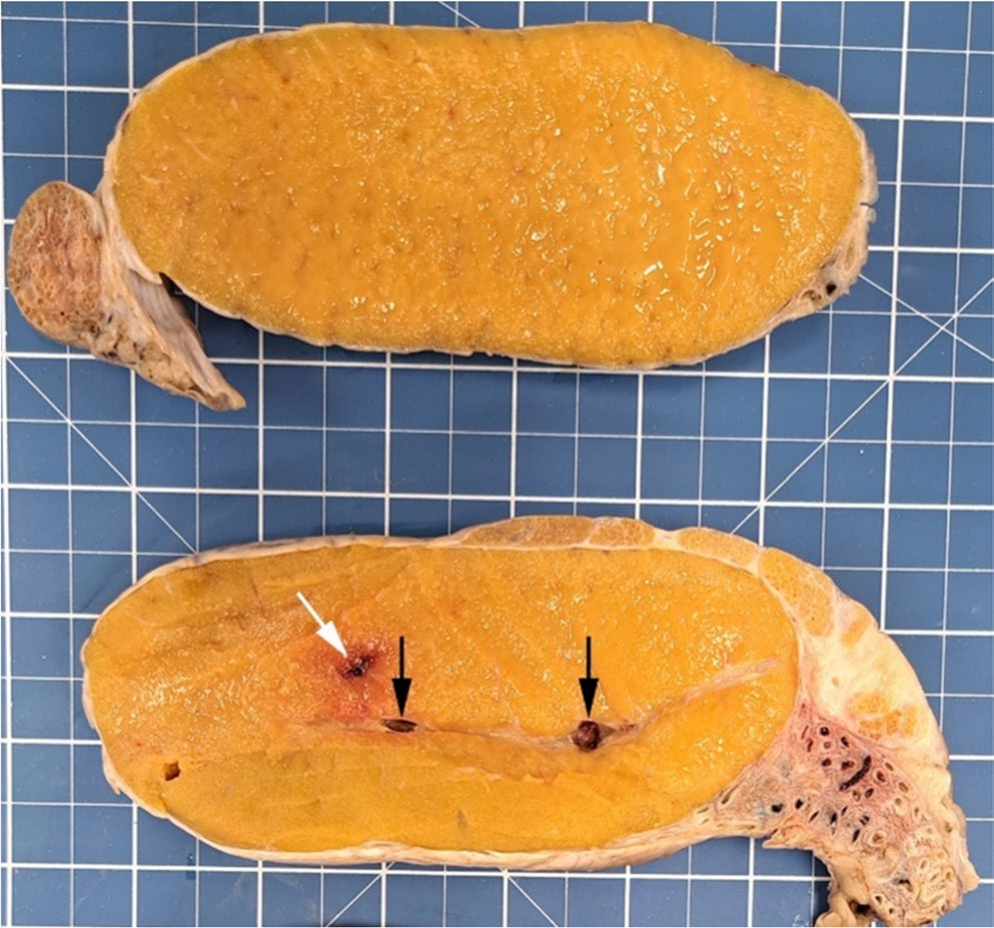
Cut surface of the left testis of a Nellore bull (*Bos indicus*), sectioned into paramedian longitudinal slice, showing dark-red nodular areas in the mediastinum (black arrows) measuring 1.2 × 0.5 cm and 0.8 × 0.5 cm, and a testicular hematoma region caused by an intratesticular anesthetic block performed prior to orchiectomy (white arrow; 15 ml lidocaine without vasoconstrictor administered using an 18G needle). (Grid squares = 1 cm.)

Grossly, the left testis measured 17.0 × 9.0 × 7.5 cm. The cut surface of the mediastinum contained two soft, dark-red nodules with cavitations, the larger measured 1.2 × 0.5 cm and the smaller 0.8 × 0.5 cm (Fig. 2).

Focal hemorrhagic changes were also observed in the testicular parenchyma and capsule and were considered procedure-related to the intratesticular anesthetic block. The epididymis, pampiniform plexus, and scrotum did not have macroscopic alterations.

### Histopathological findings

Tissue samples from the left testis, epididymis, pampiniform plexus, and scrotum were fixed in 10% buffered formalin, embedded in paraffin, and sectioned at 4 μm following standard procedures. Sections were stained with hematoxylin and eosin and examined under an Olympus CX41 microscope with a MIchrome 20 MP camera. Images were acquired using Mosaic 3.0 software.

**Fig. 3:**
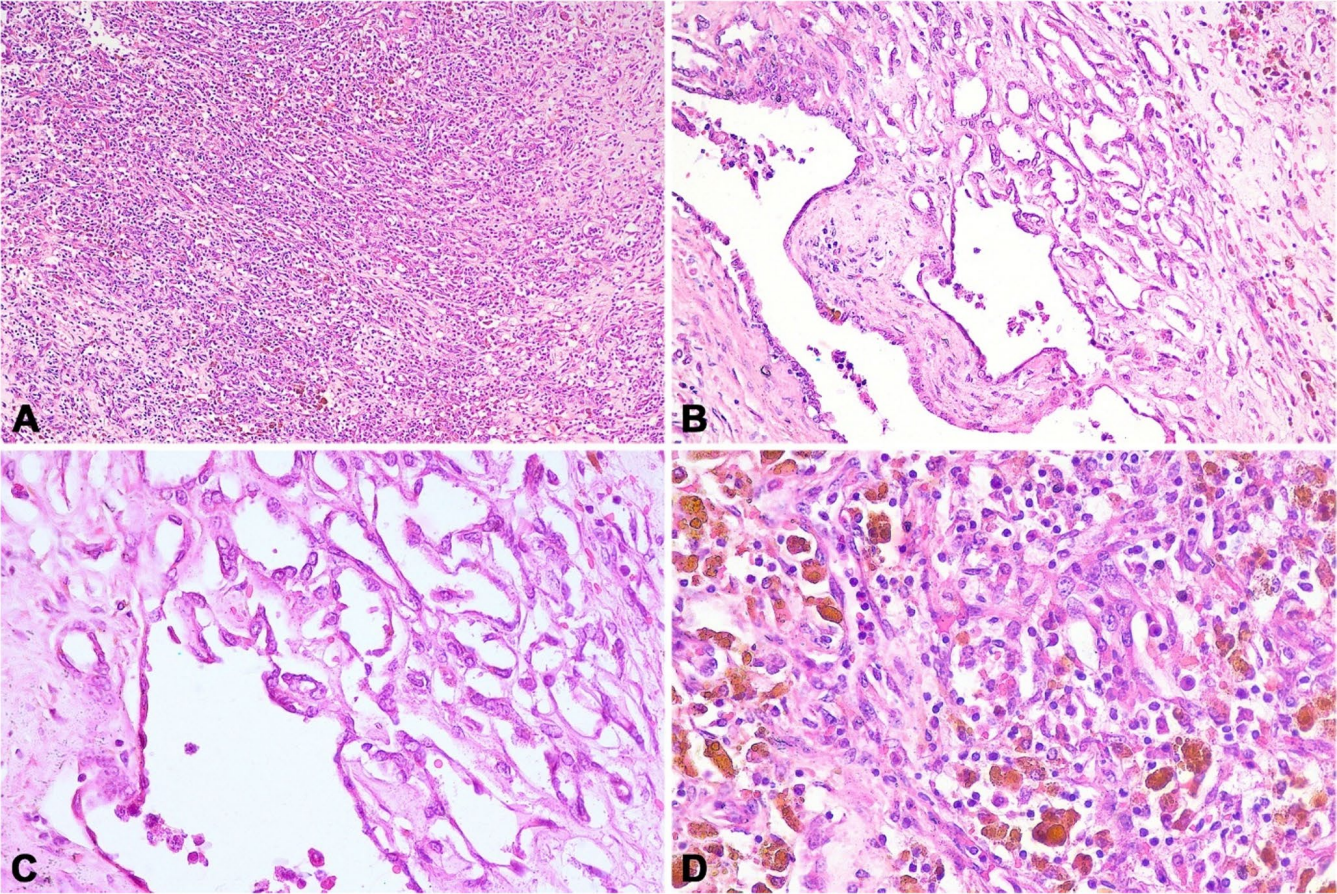
Histological findings of a carcinoma of the *rete testis* in a bull (*Bos indicus*). (**A**) Neoplastic cells arranged in a solid pattern. Hematoxylin and eosin (HE). 100X. (**B**) Neoplastic cells forming tubule-like structures of varying sizes and shapes. HE. 200X. (**C**) Neoplastic cells are cuboidal to polygonal with mild to moderate pleomorphism. HE. 400x. (**D**) Interspersed with the neoplasm are macrophages containing granular pigment and intracytoplasmic brownish pigment (hemosiderin). HE. 400X

Microscopically, the *rete testis* was infiltrated by a densely cellular, poorly demarcated, unencapsulated and infiltrative growth neoplasm confined to the mediastinum. Neoplastic cells formed solid sheets (Fig. 3A), with occasional cords supported by fibrovascular tissue. Multifocally, cells were arranged in a palisade around central lumina, forming irregular tubules (Fig. 3B, C) and papillary projections. In some areas, neoplastic cells merged with non-neoplastic *rete testis* epithelium.

Neoplastic cells were polygonal to spindle-shaped with abundant eosinophilic cytoplasm and indistinct borders (Fig. 3C). Nuclei were round to oval with finely dotted chromatin and one to two prominent nucleoli. Cells showed moderate pleomorphism, anisokaryosis, and anisocytosis, with two mitotic figures observed per 2.37 mm². Multifocal to coalescing hemorrhage and areas with hemosiderin-laden macrophages were present (Fig. 3D). About 30% of the tumor showed necrosis with eosinophilic debris. The stroma was moderately infiltrated by neutrophils, lymphocytes, and plasma cells.

**Fig. 4:**
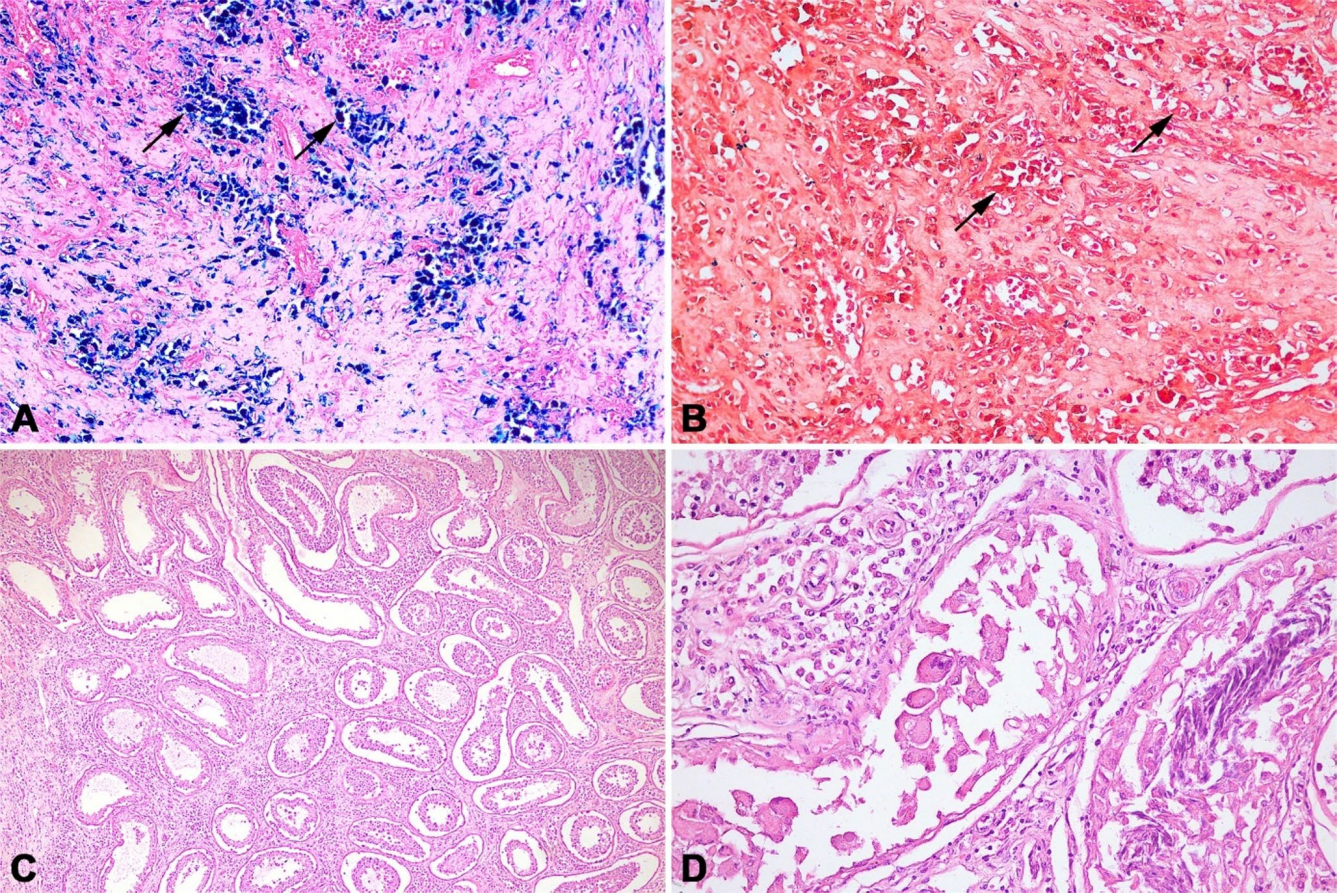
Testis of Nellore bull (*Bos indicus*) with carcinoma of the *rete testis*. (**A**) Macrophages containing intracytoplasmic pigment stained dark blue by Prussian blue, confirming it to be hemosiderin (arrows). Prussian blue. 100X. (**B**) Macrophages with brownish intracytoplasmic pigment were not reactive to the Fontana-Masson staining (arrows). Fontana-Masson. 200X. (**C**) Seminiferous tubules with decreased cellular layers and germ cells and preservation of Sertoli cells, interstitial fibrosis, and inflammatory infiltrate of lymphocytes, plasma cells, and macrophages, and some neutrophils. 40X. (**D**) Hypocellular seminiferous tubule, with multinucleated cells in the lumen. Hematoxylin and eosin. 200X

Prussian blue staining confirmed that the cytoplasmic pigment represented hemosiderin (Fig. 4A), while the pigment-containing macrophages were negative for Fontana-Masson staining, excluding melanin deposition (Fig. 4B).

In the adjacent testicular parenchyma, the seminiferous tubules were degenerate (Fig. 4C), primarily due to a decrease in the germ cell population. Most spermatids were lost, along with some spermatocytes. Numerous multinucleated spermatids were observed within the lumen of the seminiferous tubules (Fig. 4D), indicating degeneration. The interstitium showed mild to moderate multifocal infiltration with contained focal aggregates of lymphocytes, plasma cells, macrophages, and fewer neutrophils. Additionally, moderate multifocal to coalescent hemorrhage was present.

Based on histopathologic examination, the main differential diagnoses considered included epithelial neoplasms arising from the *rete testis*, mesenchymal tumors of the mediastinum testis, and vascular tumors such as hemangiosarcoma, given the mediastinal location and the presence of hemorrhage. However, the absence of well-formed vascular channels lined by atypical endothelial cells and the lack of erythrocytes within neoplastic-lined spaces made a primary vascular neoplasm less likely. Immunohistochemistry was therefore focused on confirming epithelial differentiation and excluding alternative lineages.

### Immunohistochemical findings

Immunohistochemistry was performed on formalin-fixed, paraffin-embedded sections using antibodies against pan-cytokeratin and vimentin. Sections were deparaffinized and rehydrated. Endogenous peroxidase activity was blocked with 3.5% hydrogen peroxide for 45 min. Slides were incubated with the primary antibodies under the conditions detailed in Table 1, followed by application of the appropriate detection system and visualization with chromogen EnVision Flex HRP Magenta (Dako) for 5 min. Sections were counterstained with hematoxylin. Antibody-specific details, including clones, manufacturers, dilutions, and incubation times, are summarized in Table 1.

**Table 1:** Antibodies and immunohistochemical protocol details

Antibody (clone/manufacturer)	Dilution	Primary antibody incubation	Secondary antibody
Pan-cytokeratin (AE1 AE3 - Santa Cruz Biotechnology, USA)	1:1000	Overnight at 4 °C	Anti-rabbit conjugated with peroxidase (Envisioflex HRP, Dako)
Vimentin (V9 - Dako, Denmark)	Ready to use	Overnight at 4 °C	Anti-rabbit conjugated with peroxidase (Envisioflex HRP, Dako)

**Fig. 5:**
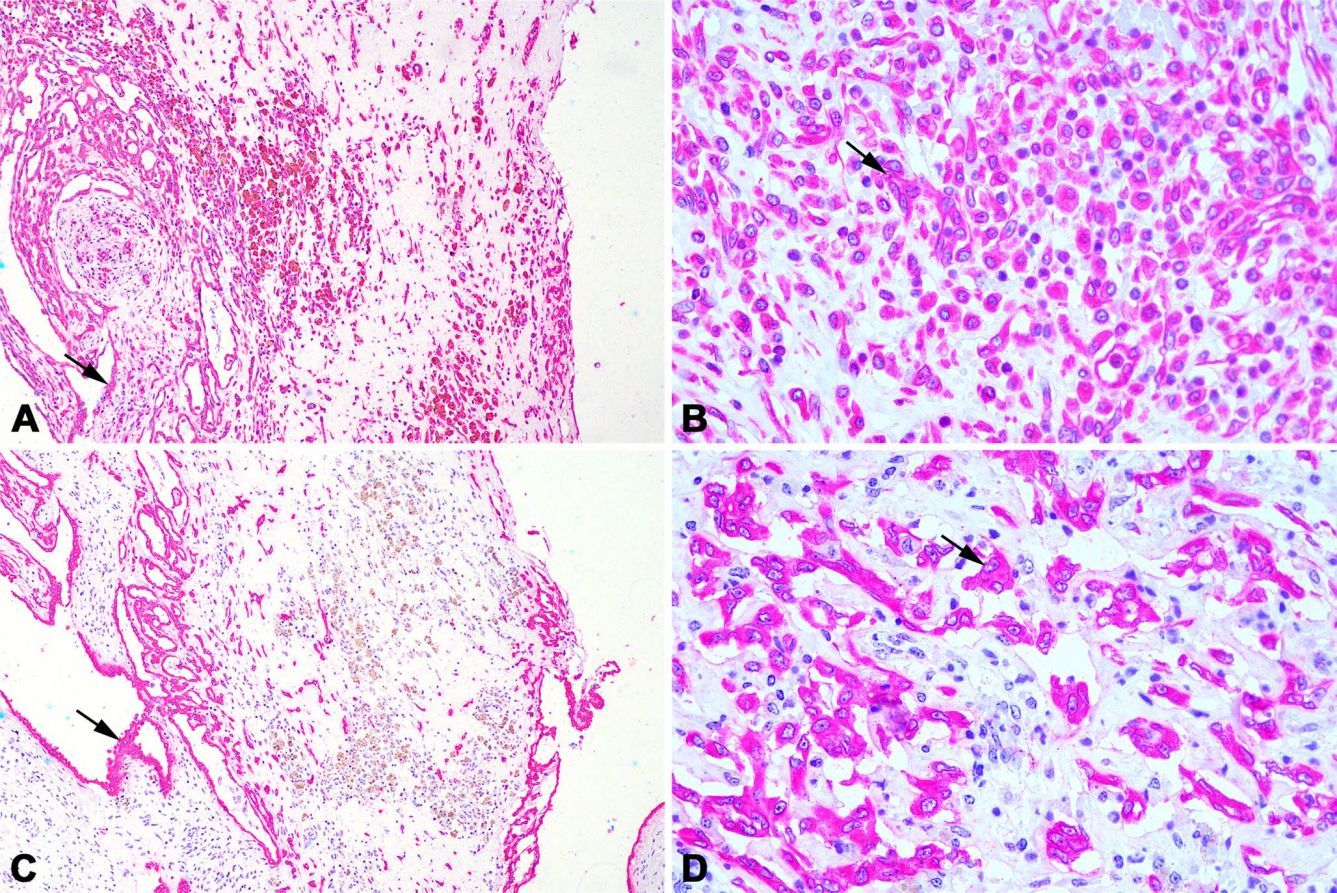
Immunohistochemistry of carcinoma of the *rete testis* in a bull (*Bos indicus*). Cytoplasmic expression of vimentin (arrows) (**A **and **B**) and pan-cytokeratin (arrows) (**C **and **D**), with positive immunoreactivity visualized as red/magenta staining. Total magnification **A**: 100X; **B**: 400x; **C**: 100X; and **D**: 400X. Visualization with EnVision Flex HRP Magenta (Dako), counterstained with hematoxylin

Internal positive controls were observed within the non-neoplastic testicular tissue, including vimentin immunoreactivity in Sertoli and interstitial cells and cytokeratin labeling in the epithelium of the *rete testis*. Neoplastic cells had marked and diffuse immunostaining for vimentin (Fig. 5A and B), and the cells were also strongly positive for cytokeratin with a multifocal to coalescing pattern (Fig. 5C and D).

**Fig. 6:**
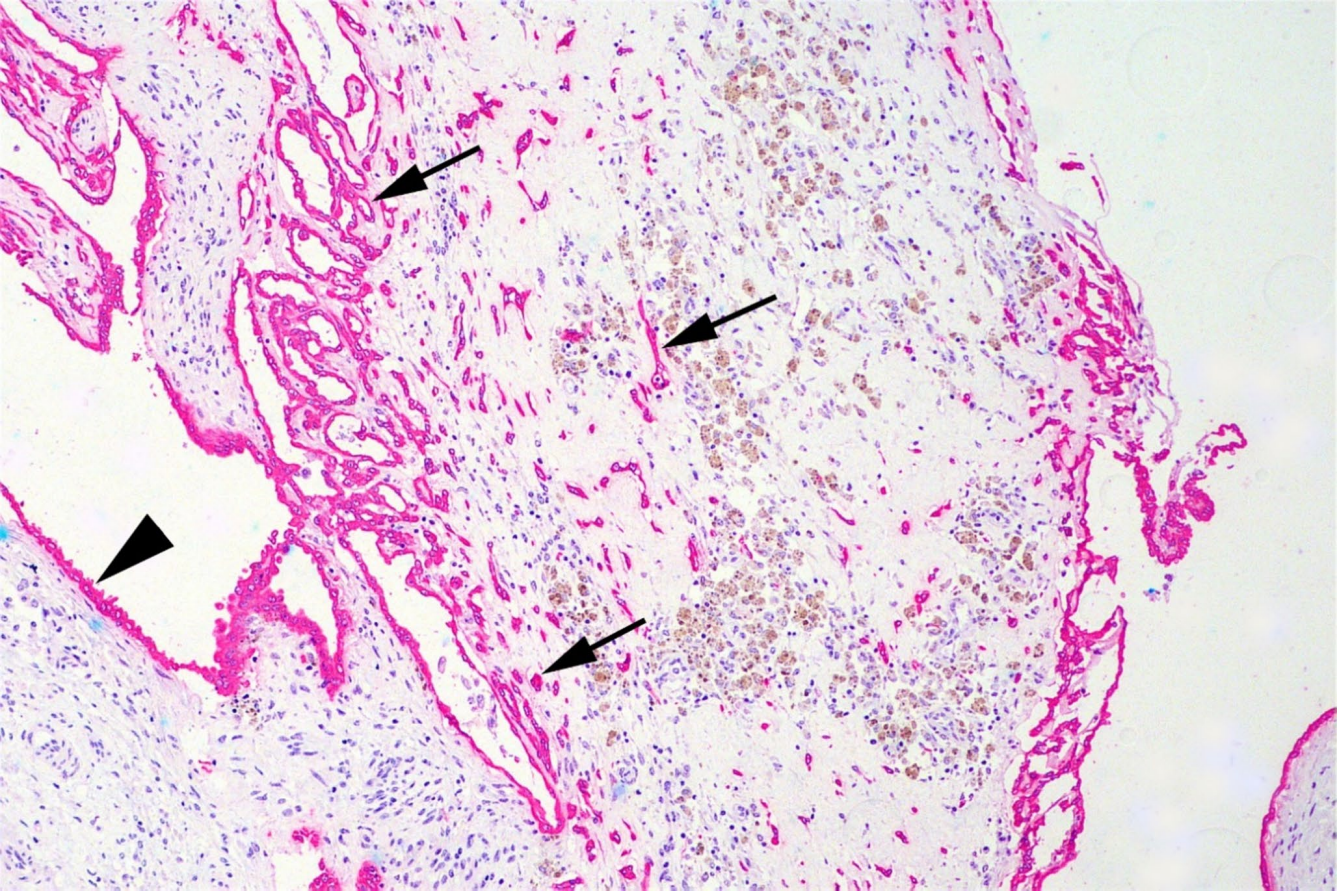
Testis of Nellore bull (*Bos indicus*) with carcinoma of the *rete testis*. Transition between normal *rete testis* epithelium (arrowhead) and neoplastic proliferation. Arrows indicate invasive growth of neoplastic cells, which are continuous with the epithelium and infiltrate the adjacent fibrovascular stroma. Cytoplasmic expression of pan-cytokeratin, with positive immunoreactivity visualized as red/magenta staining. Total magnification 100X. Visualization with EnVision Flex HRP Magenta (Dako), counterstained with hematoxylin

Histological examination revealed a transition between the normal *rete testis* epithelium and the neoplasm. The neoplastic cells were continuous with the epithelium and infiltrated the adjacent fibrovascular stroma, indicating an invasive growth pattern. Immunohistochemical staining further highlighted the distinction between normal and neoplastic tissues, facilitating the identification of the transition and supporting the interpretation of the carcinoma (Fig. 6).

### Diagnosis and interpretation

Based on morphologic features, including the location restricted to the mediastinum and contiguous to the *rete testis* epithelium, and co-expression of both vimentin and cytokeratin by neoplastic cells, the findings supported the diagnosis of primary carcinoma of the *rete testis*.

## Discussion and conclusions

No previous reports were found of carcinoma of the *rete testis* in bulls. In humans, this tumor is exceedingly rare, aggressive, and associated with a poor prognosis, with approximately 80 cases reported worldwide (Maganty et al. 2018; Kitano et al. 2020; Mitchell and Pearce 2022; Petrović et al. 2023; Li et al. 2024; Chen et al. 2025). The clinicopathological presentation in this case included hemospermia, cystic lesions in the mediastinum testis on ultrasound, and retention of reproductive function following unilateral orchiectomy. The gradual transition between normal and neoplastic *rete testis* epithelium observed in this bull supports the identification of this lesion as a primary carcinoma of the *rete testis* (Radi et al. 2004; Al-Obaidy et al. 2019; Suarez-Zamora et al. 2021).

The immunohistochemical profile of the neoplasm showed co-expression of pancytokeratin and vimentin. This staining profile is well recognized in carcinoma of the *rete testis* (Radi et al. 2004; Al-Obaidy et al. 2019). This feature aligns with the embryologic origin of the *rete testis* from mesonephric and mesothelial-associated structures, which retain a hybrid phenotype with both epithelial and mesenchymal attributes. Accordingly, cytokeratin expression denotes epithelial differentiation, whereas vimentin positivity corresponds to similarities with mesonephric and mesothelial tissues reported in human cases (Suarez-Zamora et al. 2021; Chen et al. 2025). In the largest aggregated series, AE1/AE3 cytokeratin is positive in ~ 96% of cases, with frequent co-positivity for EMA (~ 84%), CK7 (~ 83%), and vimentin (~ 81%) (Al-Obaidy et al. 2019). This immunophenotypic combination assists in distinguishing *rete testis* carcinoma from germ cell tumors, sex cord-stromal tumors and metastatic carcinomas, which typically lack this pattern of dual epithelial-mesenchymal expression (Huang and Chang 2015; Suarez-Zamora et al. 2021). Taken together, cytokeratin-vimentin co-expression strongly supports the diagnosis and reflects the expected immunobiological behavior of tumors arising from this embryologic region (Agnew and MacLachlan 2017).

Differential diagnoses for *rete testis* tumors include neoplasms with overlapping morphological features, such as malignant mesothelioma of the vaginal tunics, sustentacular cell tumors, seminomas and teratomas located near the mediastinum, metastatic carcinomas, as well as inflammatory and infectious conditions (Radi et al. 2004; Chekol and Sun 2012; Huang and Chang 2015; Agnew and MacLachlan 2017; Suarez-Zamora et al. 2021; Petrović et al. 2023). Diagnostic criteria established in human pathology and reported in animals include the location in the mediastinum, absence of other primary neoplasms, morphology incompatible with alternative tumor types, and evidence of transition from normal to neoplastic rete testis epithelium (Radi et al. 2004; Chen et al. 2025). In distinguishing carcinoma from non-neoplastic proliferations of the *rete testis*, hyperplasia presents as an orderly epithelial proliferation without atypia or expansive masses; adenoma forms well-circumscribed, expansile lesions with absent or minimal atypia; while carcinoma demonstrates infiltrative growth, destruction of adjacent stroma, and moderate to marked cytologic atypia, sometimes with necrosis.

Regarding hemospermia, no previous reports have linked it specifically to carcinoma of the *rete testis*. Moreover, this finding is considered rare in human patients with genitourinary neoplasms (0.21%) and is more frequently associated with prostatic tumors (Efesoy et al. 2021). However, Efesoy et al. (2021) described hemospermia associated with multiple testicular and paratesticular neoplasms confirmed by histopathological analysis, including seminoma and non-germ cell tumors. These were categorized into tumors confined to the testis with lymphovascular and *rete testis* invasion, tumors confined to the testis without *rete testis* invasion, and tumors involving both the testis and epididymis, with vaginal tunics infiltration. These findings support the present case, suggesting that hemospermia may reflect the extent of the neoplasm and its tissue invasion characteristics.

Inflammation in *rete testis* carcinoma is generally interpreted as a secondary reaction to tissue disruption caused by the expanding neoplasm, particularly when cystic dilation or stromal invasion is present. Human reports similarly describe peritumoral inflammation as a reactive rather than intrinsic neoplastic feature (Huang and Chang 2015; Suarez-Zamora et al. 2021). In the present case, the inflammatory infiltrate surrounding the lesion likely reflects local epithelial damage, ductal obstruction, and degenerative changes associated with progressive cystic expansion, or a response to tumor products, such as cytokeratin.

In conclusion, the diagnosis of carcinoma of the *rete testis* was established based on the anatomic location, histopathologic features, and immunohistochemical profile compatible with both epithelial and mesenchymal markers. Although rare, this neoplasm should be considered as a differential diagnosis in bulls presenting with hemospermia or intratesticular cystic lesions. Early recognition is essential for guiding reproductive management and preserving fertility, and the integration of imaging, histopathology, and immunohistochemistry enhances diagnostic accuracy for such uncommon testicular tumors.

## Data Availability

No datasets were generated or analysed during the current study.
